# ﻿*Phaeotubakialithocarpicola* gen. et sp. nov. (Tubakiaceae, Diaporthales) from leaf spots in China

**DOI:** 10.3897/mycokeys.95.98384

**Published:** 2023-01-09

**Authors:** Ning Jiang, Ya-Quan Zhu, Han Xue, Chun-Gen Piao, Yong Li

**Affiliations:** 1 Key Laboratory of Biodiversity Conservation of National Forestry and Grassland Administration, Ecology and Nature Conservation Institute, Chinese Academy of Forestry, Beijing 100091, China Ecology and Nature Conservation Institute, Chinese Academy of Forestry Beijing China

**Keywords:** *Ascomycota*, morphology, new genus, phylogeny, plant disease, taxonomy, Tubakiaceae

## Abstract

Tubakiaceae represents a distinct lineage of Diaporthales, including its type genus *Tubakia* and nine additional known genera. Tubakiaceous species are commonly known as endophytes in leaves and twigs of many tree species, but can also be plant pathogens causing conspicuous leaf symptoms. In the present study, isolates were obtained from diseased leaves of *Lithocarpusglaber* collected in Guangdong Province, China. The identification was conducted based on morphology and phylogeny of combined loci of 28S nrRNA gene (LSU), internal transcribed spacer regions and intervening 5.8S nrRNA gene (ITS) of the nrDNA operon, translation elongation factor 1-alpha (*tef1*) and beta tubulin (*tub2*). As a result, a distinct clade in Tubakiaceae was revealed named *Phaeotubakialithocarpicola***gen. et sp. nov.**, which was distinguished from the other tubakiaceous taxa by its dark brown conidiogenous cells and conidia.

## ﻿Introduction

The fungal order Diaporthales contains members usually inhabiting plant tissues as pathogens, endophytes and saprophytes (Rossman et al. 2007; Senanayake et al. 2017, 2018; Fan et al. 2018; Jiang et al. 2021a; Udayanga et al. 2021). Tubakiaceae was proposed as a diaporthalean family based on its type genus *Tubakia*, and the other seven genera, namely *Apiognomonioides*, *Involutiscutellula*, *Oblongisporothyrium*, *Paratubakia*, *Racheliella*, *Saprothyrium* and *Sphaerosporithyrium* (Braun et al. 2018). Subsequently, *Ellipsoidisporodochium* and *Obovoideisporodochium* were added to this family based on morphological and phylogenetical evidence (Zhang et al. 2021; Liu et al. 2022). Hence, ten genera have been accepted in Tubakiaceae before the present study.

Species of Tubakiaceae are usually characterized by forming pycnothyria composed of convex scutella with radiating threads of cells fixed to the substratum by a central columella, mostly surrounded by a sheath of small fertile cells that give rise to one-celled, phialidic conidiogenous cells (Harrington et al. 2012; Braun et al. 2018). However, some species also form crustose or pustulate pycnidioid conidiomata, for example, *Tubakiacalifornica* is known to only have crustose pycnidioid conidiomata during its lifecycle (Braun et al. 2018). Moreover, conidia of tubakiaceous species are globose, subglobose, ellipsoid, broad ellipsoid-obovoid to subcylindrical or somewhat irregular in shape, aseptate, hyaline, subhyaline to pigmented (Braun et al. 2018; Zhang et al. 2021). Conidia of *Apiognomonioides*, *Ellipsoidisporodochium*, *Oblongisporothyrium*, *Obovoideisporodochium* and *Saprothyrium* species are known to be hyaline (Braun et al. 2018; Zhang et al. 2021; Liu et al. 2022). Conidia of *Involutiscutellula*, *Paratubakia* and *Sphaerosporithyrium* species are hyaline to slightly pigmented (Braun et al. 2018), while conidia of *Racheliella* and *Tubakia* species are hyaline to pigmented (Braun et al. 2014, 2018; Zhu et al. 2022).

Tubakiaceae species are known to be endophytes in leaves and twigs of many tree species, but can also cause conspicuous symptoms on host leaves as plant pathogens (Harrington et al. 2012; Braun et al. 2018; Zhu et al. 2022). Nearly all tubakiaceous species are reported from Fagaceae, such as species of *Castanea*, *Castanopsis*, *Fagus*, *Lithocarpus* and *Quercus* (Braun et al. 2018; Morales-Rodríguez et al. 2021). In addition, these fungi are also discovered from the other plant families, i.e., Altingiaceae, Anacardiaceae, Nyssaceae, Oleaceae, Rosaceae, Sapindaceae and Ulmaceae (Braun et al. 2018; Liu et al. 2022).

The aim of the present study is to identify two isolates obtained from diseased leaves of *Lithocarpusglaber* from Guangdong Province by morphological characters and phylogeny based on combined loci of 28S nrRNA gene (LSU), internal transcribed spacer regions and intervening 5.8S nrRNA gene (ITS) of the nrDNA operon, translation elongation factor 1-alpha (*tef1*) and beta tubulin (*tub2*).

## ﻿Materials and methods

### ﻿Sample collection, fungal isolation and morphology

Diseased leaves of *Lithocarpusglaber* were collected from Guangdong Province, China. The leaf samples were packed in paper bags and transferred to the laboratory for isolation. The leaves were firstly surface-sterilized for 2 min in 75% ethanol, 4 min in 1.25% sodium hypochlorite, and 1 min in 75% ethanol, then rinsed for 2 min in distilled water and blotted on dry sterile filter paper. Then diseased tissues were cut into 0.5 cm × 0.5 cm pieces using a double-edge blade, and transferred onto the surface of potato dextrose agar (PDA, 200 g potatoes, 20 g dextrose, 20 g agar per L), and incubated at 25 °C to obtain cultures. The hyphal tips were then transferred to clean plates of PDA, malt extract agar (MEA, 30 g malt extract, 5 g mycological peptone, 15 g agar per L) and synthetic low nutrient agar (SNA, 1 g KN2PO4, 1 g KNO3, 0.5 g MgSO4-7H2O, 0.5 g KCl, 0.2 g glucose, 0.5 g gucrose per L) under a dissecting stereomicroscope with sterile needles. The cultures were deposited in China Forestry Culture Collection Center (CFCC, http://cfcc.caf.ac.cn/; accessed on 6 December 2022), and the specimens in the herbarium of the Chinese Academy of Forestry (CAF, http://museum.caf.ac.cn/; accessed on 6 December 2022).

Morphology of the new taxa was studied based on conidiomata formed on PDA plates under a dissecting microscope (M205 C, Leica, Wetzlar, Germany). The conidiogenous cells and conidia were immersed in tap water, then the microscopic photographs were captured with an Axio Imager 2 microscope (Zeiss, Oberkochen, Germany) equipped with an Axiocam 506 color camera, using differential interference contrast (DIC) illumination. More than 50 conidia were randomly selected for measurement. Culture characters were recorded from PDA, MEA and SNA after 10 days at 25 °C in the dark.

### ﻿DNA extraction, PCR amplification and phylogenetic analyses

The fungal genomic DNA was extracted from mycelia grown on PDA palates after 10 days following the method in Doyle and Doyle (1990). Four partial loci, ITS and LSU regions, *tef1* and *tub2* genes were amplified by the following primer pairs: ITS1 and ITS4 for ITS (White et al. 1990), LR0R and LR5 for LSU (Vilgalys and Hester 1990), EF1-688F and EF2 for *tef1* (Carbone and Kohn 1999), and Bt2a and Bt2b for *tub2* (Glass and Donaldson 1995).

The polymerase chain reaction (PCR) conditions were set as follows: an initial denaturation step of 5 min at 94 °C, followed by 35 cycles of 30 s at 94 °C, 50 s at 48 °C (ITS and LSU) or 54 °C (*tef1* and *tub2*), and 1 min at 72 °C, and a final elongation step of 10 min at 72 °C. PCR products were assayed via electrophoresis in 2% agarose gels. DNA sequencing was performed using an ABI PRISM 3730XL DNA Analyser with a BigDye Terminator Kit v.3.1 (Invitrogen, Waltham, MA, USA) at the Shanghai Invitrogen Biological Technology Company Limited (Beijing, China).

The sequences obtained in the current study were assembled using SeqMan v. 7.1.0, and reference sequences were retrieved from the website of the National Center for Biotechnology Information (NCBI, https://www.ncbi.nlm.nih.gov; accessed on 15 October 2022), based on sequences from Braun et al. (2018) and Zhang et al. (2021). The sequences were aligned using MAFFT v. 7 and corrected manually using MEGA v. 7.0.21 (Katoh et al. 2019).

The phylogenetic analyses of combined matrixes of ITS-LSU-*tef1*-*rpb2* were performed using maximum parsimony (MP), maximum likelihood (ML) and Bayesian inference (BI) methods. MP analysis was run using a heuristic search option of 1000 search replicates with random-additions of sequences with a tree bisection and reconnection (TBR) algorithm in PAUP v. 4.0b10 (Swofford 2003). Maxtrees were set to 5 000, branches of zero length were collapsed and all equally parsimonious trees were saved. Other calculated parsimony scores were tree length (TL), consistency index (CI), retention index (RI) and rescaled consistency (RC). ML was implemented on the CIPRES Science Gateway portal (https://www.phylo.org) using RAxML-HPC BlackBox 8.2.10 (Miller et al. 2010; Stamatakis 2014), employing a GTR-GAMMA substitution model with 1000 bootstrap replicates. Bayesian inference was performed using a Markov Chain Monte Carlo (MCMC) algorithm in MrBayes v. 3.0 (Ronquist and Huelsenbeck 2003). Two MCMC chains, starting from random trees for 1000000 generations and trees, were sampled every 100^th^ generation, resulting in a total of 10000 trees. The first 25% of trees were discarded as burn-in of each analysis. Branches with significant Bayesian Posterior Probabilities (BPP > 0.9) were estimated in the remaining 7500 trees. Phylogenetic trees were viewed with FigTree v. 1.4.2 and processed by Adobe Illustrator CS5. The nucleotide sequence data of the new taxa were deposited in GenBank, and the GenBank accession numbers of all accessions included in the phylogenetic analyses are listed in Table 1.

**Table 1.** Isolates and GenBank accession numbers used in the phylogenetic analyses.

Species	Isolate^a^	Host	Location	GenBank accession number			
				ITS	LSU	*tef1*	*tub2*
*Apiognomonioidessupraseptata*	CBS 632.92*	*Quercusglauca*	Japan	MG976447	MG976448	NA	NA
*Ellipsoidisporodochiumphotiniae*	SAUCC 210421*	*Photiniaserratifolia*	China	OK175559	OK189532	OK206440	OK206442
*Ellipsoidisporodochiumphotiniae*	SAUCC 210423	*Photiniaserratifolia*	China	OK175560	OK189533	OK206441	OK206443
*Involutiscutellularubra*	CBS 192.71*	*Quercusphillyraeoides*	Japan	MG591899	MG591993	MG592086	MG592180
*Involutiscutellularubra*	MUCC2303	*Quercusphillyraeoides*	Japan	MG591900	MG591994	MG592087	MG592181
*Involutiscutellularubra*	MUCC2305	*Quercusphillyraeoides*	Japan	MG591902	MG591996	MG592089	MG592182
*Melanconisgroenlandica*	CBS 116540*	*Betulanana*	Greenland	KU878552	KU878553	KU878554	KU878555
*Oblongisporothyriumcastanopsidis*	CBS 124732	*Castanopsiscuspidata*	Japan	MG591849	MG591942	MG592037	MG592131
*Oblongisporothyriumcastanopsidis*	CBS 189.71*	*Castanopsiscuspidata*	Japan	MG591850	MG591943	MG592038	MG592132
*Obovoideisporodochiumlithocarpi*	SAUCC 0748*	*Lithocarpusfohaiensis*	China	MW820279	MW821346	MZ996876	MZ962157
*Paratubakiasubglobosa*	CBS 124733	*Quercusglauca*	Japan	MG591913	MG592008	MG592102	MG592194
*Paratubakiasubglobosa*	CBS 193.71*	*Quercusglauca*	Japan	MG591914	MG592009	MG592103	MG592195
*Paratubakiasubglobosoides*	MUCC2293*	*Quercusglauca*	Japan	MG591915	MG592010	MG592104	MG592196
***Phaeotubakialithocarpicola***	CFCC 54422*	***Lithocarpusglaber***	**China**	**OP951017**	**OP951015**	**OQ127584**	**OQ127586**
***Phaeotubakialithocarpicola***	**RK7CX**	***Lithocarpusglaber***	**China**	**OP951018**	**OP951016**	**OQ127585**	**OQ127587**
*Racheliellawingfieldiana*	CBS 143669*	*Syzigiumguineense*	South Africa	MG591911	MG592006	MG592100	MG592192
*Saprothyriumthailandense*	MFLUCC 12-0303*	Decaying leaf	Thailand	MF190163	MF190110	NA	NA
*Sphaerosporithyriummexicanum*	CPC 31361	*Quercuseduardi*	Mexico	MG591894	MG591988	MG592081	MG592175
*Sphaerosporithyriummexicanum*	CPC 32258	*Quercuseduardi*	Mexico	MG591895	MG591989	MG592082	MG592176
*Sphaerosporithyriummexicanum*	CPC 33021*	*Quercuseduardi*	Mexico	MG591896	MG591990	MG592083	MG592177
*Tubakiaamericana*	CBS 129014	*Quercusmacrocarpa*	USA	MG591873	MG591966	MG592058	MG592152
*Tubakiacalifornica*	CPC 31496	*Quercusagrifolia*	USA	MG591829	MG591922	MG592017	MG592111
*Tubakiacalifornica*	CPC 31499	*Quercuswislizeni*	USA	MG591832	MG591925	MG592020	MG592114
*Tubakiadryina*	CBS 112097*	*Quercusrobur*	Italy	MG591851	MG591944	MG592039	MG592133
*Tubakiadryina*	CBS 114912	*Quercus* sp.	Netherlands	MG591853	MG591946	MG592041	MG592135
*Tubakiadryina*	CBS 129016	*Quercusalba*	USA	MG591870	MG591963	MG592056	MG592150
*Tubakiadryinoides*	CBS 329.75	*Quercus* sp.	France	MG591874	MG591967	MG592059	MG592153
*Tubakiadryinoides*	CBS 190.71	*Castaneacrenata*	Japan	MG591876	MG591968	MG592061	MG592155
*Tubakiahallii*	CBS 129013*	*Quercusstellata*	USA	MG591880	MG591972	MG592065	MG592159
*Tubakiahallii*	CBS 129015	*Quercusstellata*	USA	MG591881	MG591973	MG592066	MG592160
*Tubakiajaponica*	CBS 191.71	*Castaneacrenata*	Japan	MG591885	MG591977	MG592070	MG592164
*Tubakialiquidambaris*	CBS 139744	*Liquidambarstyraciflua*	USA	MG605068	MG605077	MG603578	NA
*Tubakiamelnikiana*	CPC 32249	*Quercuscanbyi*	Mexico	MG591889	MG591983	MG592076	MG592170
*Tubakiaoblongispora*	MUCC2295*	*Quercusserrata*	Japan	MG591897	MG591991	MG592084	MG592178
*Tubakiaparadryinoides*	MUCC2294*	*Quercusacutissima*	Japan	MG591898	MG591992	MG592085	MG592179

Note: NA, not applicable. Ex-type strains are marked with *, and strains from the present study are in black bold. ^a^ CBS: Westerdijk Fungal Biodiversity Institute, Utrecht, the Netherlands; CFCC: China Forestry Culture Collection Center, Beijing, China; CPC: Culture collection of P. W. Crous, housed at CBS; MFLUCC: Mae Fah Luang University Culture Collection, Thailand; MUCC: Lab. of Plant Pathology, Mie University, Japan; SAUCC: Shandong Agricultural University Culture Collection, China.

## ﻿Results

### ﻿Phylogenetic analyses

**Figure 1.**
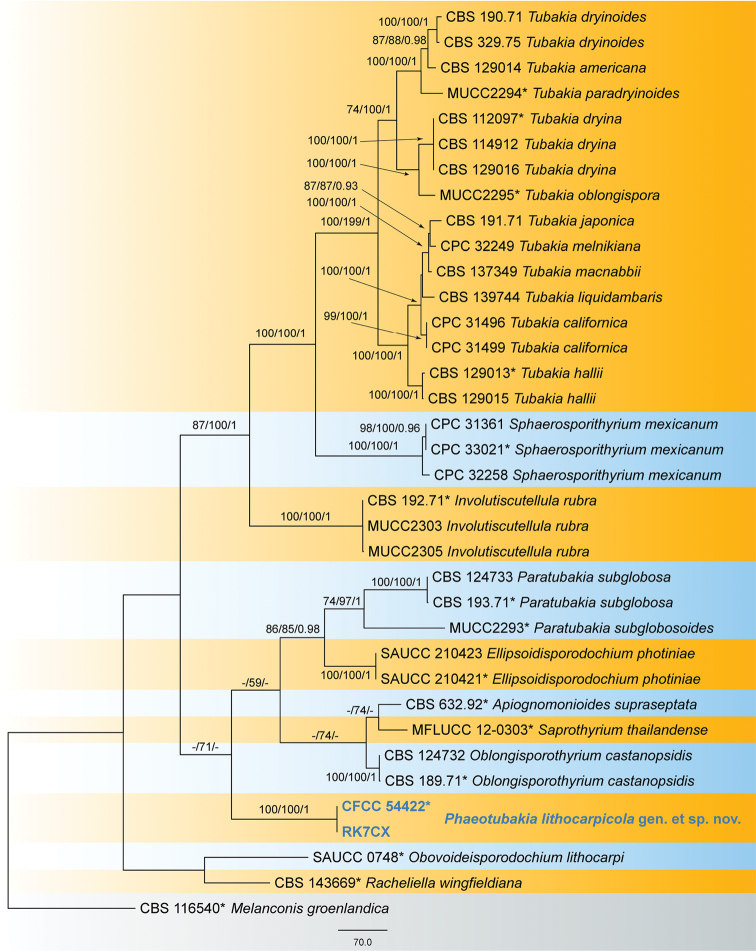
Phylogram of Tubakiaceae based on combined ITS, LSU, *tef1* and *tub2* loci. Numbers above the branches indicate maximum parsimony bootstrap (MP BP ≥ 50%), ML bootstrap values (ML-BS ≥ 50%) and Bayesian Posterior Probabilities (BPP ≥ 0.9). The tree is rooted with *Melanconisgroenlandica* (CBS 116540). Ex-type strains are marked with *, and strains from the present study are marked in bold blue.

The alignment based on the sequence dataset (ITS, LSU, *tef1* and *tub2*) included 35 ingroup taxa, comprising 2736 characters in the aligned matrix. Of these, 1721 characters were constant, 206 variable characters were parsimony-uninformative and 809 characters were parsimony informative. The MP analysis resulted in two equally most parsimonious trees (TL = 2708, CI = 0.615, RI = 0.804, RC = 0.385) and the first tree is shown in Fig. 1. The topologies resulting from MP, ML and BI analyses of the concatenated dataset were congruent. Isolates from the present study formed an individual clade in Tubakiaceae representing a new genus and species named *Phaeotubakialithocarpicola*.

### ﻿Taxonomy

#### ﻿ Phaeotubakia

Taxon classificationFungiDiaporthalesTubakiaceae

Ning Jiang gen. nov.

24795432-F4C2-5879-85F3-C7B99BE789D1

 MB846813

##### Etymology.

Named derived from *phaeo* (= pigmented) and its morphological similarity to *Tubakia*.

##### Type species.

*Phaeotubakialithocarpicola* Y.Q. Zhu & Ning Jiang.

##### Description.

Sexual morph: Unknown. Asexual morph in vitro: Conidiomata sporodochial, slimy, black, semi-submerged. Conidiophores reduced to conidiogenous cells. Conidiogenous cells brown, smooth, guttulate, cylindrical to ampulliform, attenuate towards apex, phialidic. Conidia blastic, subglobose, broad ellipsoid to ellipsoid, seldom irregular, brown to dark brown, walls smooth, becoming thicker with age, base rounded or with truncate basal hilum.

##### Notes.

*Phaeotubakia* is proposed as the eleventh genus of Tubakiaceae based on morphological features and phylogeny of combined ITS, LSU, *tef1* and *tub2* loci (Fig. 1). *Phaeotubakia* is distinguished from *Apiognomonioides*, *Ellipsoidisporodochium*, *Involutiscutellula*, *Oblongisporothyrium*, *Obovoideisporodochium*, *Paratubakia*, *Racheliella*, *Saprothyrium* and *Sphaerosporithyrium* by having brown to dark brown conidia (Braun et al. 2018; Zhang et al. 2021). Several species of *Tubakia* are known to have brown conidia, which is similar to *Phaeotubakialithocarpicola* (Braun et al. 2018; Zhu et al. 2022). However, they are phylogenetically distinct (Fig. 1).

#### ﻿ Phaeotubakia lithocarpicola

Taxon classificationFungiDiaporthalesTubakiaceae

Y.Q. Zhu & Ning Jiang sp. nov.

3033F3C5-37E1-5D83-910E-65A6A9188207

 MB846814

Fig. 2

##### Etymology.

Named after the host genus, *Lithocarpus*.

##### Description.

From leaf spots, circular to subcircular, margin distinct, brown to fuscous. Sexual morph: Unknown. Asexual morph in vitro: Conidiomata sporodochial, appeared after 10 days on PDA surface, slimy, black, semi-submerged, 50–350 μm diam. Conidiophores reduced to conidiogenous cells. Conidiogenous cells brown, smooth, guttulate, cylindrical to ampulliform, attenuate towards apex, phialidic, 6–15.5 × 3.5–5 μm. Conidia blastic, subglobose, broad ellipsoid to ellipsoid, seldom irregular, brown to dark brown, walls smooth, becoming thicker with age, base rounded or with truncate basal hilum, (13.5–)14–16.5(–18) × (5.5–)7–8.5(–9) μm (n = 50), L/W = 1.7–3.2.

**Figure 2.**
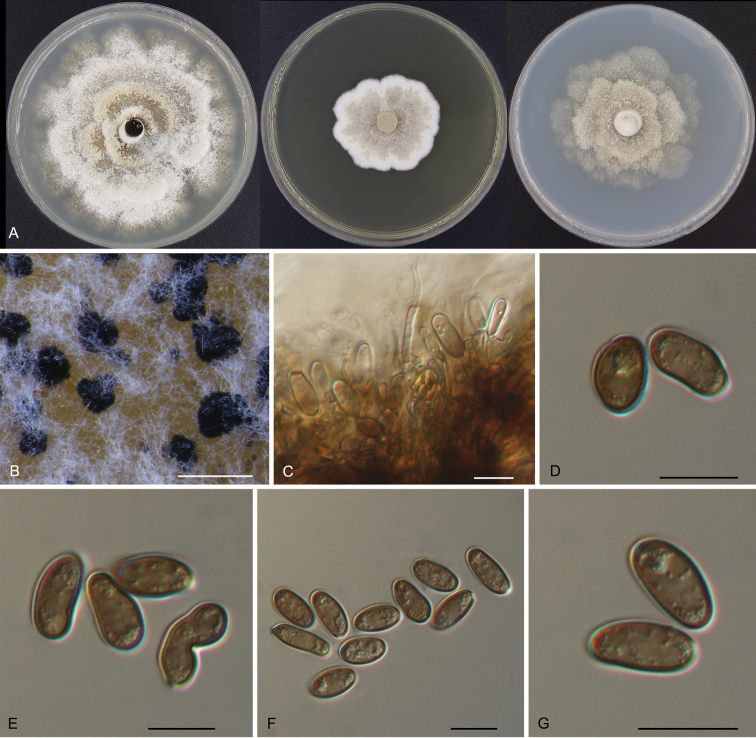
Morphology of *Phaeotubakialithocarpicola* (CFCC 54452) **A** colonies on PDA, MEA and SNA after 10 days at 25 °C **B** conidiomata formed on PDA**C** conidiogenous cells giving rise to conidia **D–G** conidia. Scale bars: 200 μm (**B**); 10 μm (**C–G**).

##### Culture characters.

Colonies on PDA flat, spreading, with flocculent aerial mycelium, white to pale luteous, with age forming concentric zones, reaching a 90 mm diameter and forming abundant black conidiomata after 10 days at 25 °C; on MEA flat, spreading, with flocculent aerial mycelium and crenate edge, pale luteous to pale grey, reaching a 45 mm diameter after 10 days at 25 °C; on SNA flat, spreading, with flocculent aerial mycelium forming concentric rings and entire edge, pale luteous, reaching a 60 mm diameter after 10 days at 25 °C.

##### Specimens examined.

China, Guangdong Province, Qingyuan City, Yangshan County, Guangdong Nanling Nature Reserve, on diseased leaves of *Lithocarpusglaber*, 4 December 2019, Yong Li (holotype CAF 800071; ex-holotype culture CFCC 54422). Guangdong Province, Qingyuan City, Yangshan County, Guangdong Nanling Nature Reserve, on diseased leaves of *Lithocarpusglaber*, 3 December 2019, Dan-ran Bian (culture RK7CX).

##### Notes.

*Phaeotubakialithocarpicola* is the sole species within the newly proposed genus, which is associated with leaf spot disease of *Lithocarpusglaber*. Two tubakiaceous species were reported from the host genus *Lithocarpus* before the present study, viz. *Obovoideisporodochiumlithocarpi* from *Lithocarpusfohaiensis* in China and *Tubakiacalifornica* from *Lithocarpusdensiflorus* in the USA (Braun et al. 2018; Zhang et al. 2021). *Phaeotubakialithocarpicola* represents the third tubakiaceous species discovered from the host genus *Lithocarpus*. However, *P.lithocarpicola* differs from *O.lithocarpi* and *T.californica* by brown conidiogenous cells and brown to dark brown conidia (Braun et al. 2018; Zhang et al. 2021).

## ﻿Discussion

Diaporthales is a well-resolved fungal order based on evidence of both morphology and phylogeny (Senanayake et al. 2017, 2018; Fan et al. 2018; Jiang et al. 2020). *Tubakia* was placed in Melanconiellaceae of Diaporthales (Senanayake et al. 2017), and subsequently transferred to the newly established family of its own Tubakiaceae (Braun et al. 2018). Meanwhile, some species were removed from *Tubakia*, and seven new genera were proposed based on these species (Braun et al. 2018). Soon after, *Ellipsoidisporodochium* and *Obovoideisporodochium* were added to Tubakiaceae (Zhang et al. 2021; Liu et al. 2022). In the present study, the eleventh genus *Phaeotubakia* is proposed to be included in this family.

Members of Tubakiaceae are quite similar in morphology, but phylogenetically distinct (Braun et al. 2018; Senanayake et al. 2018; Zhang et al. 2021). The sexual morph of Tubakiaceae is not prominent, hence genera and species are distinguished mainly based on their asexual morphology and molecular data.

The newly proposed genus and species *Phaeotubakialithocarpicola* in the present study produce brown to dark brown conidia on the PDA plates, which is morphologically different from the other tubakiaceous taxa, but similar to *Melanconis*-like taxa of Diaporthales (Voglmayr et al. 2012, 2017; Jiang et al. 2021b). Four families of Diaporthales are known to contain *Melanconis*-like genera and species, namely Juglanconidaceae, Melanconidaceae, Melanconiellaceae and Pseudomelanconidaceae (Jiang et al. 2018; Fan et al. 2018; Senanayake et al. 2018). Hence, traditional morphological identification of diaporthalean fungi is insufficient.

The center of genetic diversity of *Tubakia* appears to be in East Asia, e.g. China and Japan, where Fagaceae hosts are the most common hosts (Harrington and McNew 2018). *Obovoideisporodochiumlithocarpi* and several new *Tubakia* species (*T.cyclobalanopsidis* and *T.quercicola*) recently discovered from trees of Fagaceae (Zhang et al. 2021; Zhu et al. 2022), and *Phaeotubakialithocarpicola* proposed in the present study support this phenomenon well. More taxa of Tubakiaceae may be revealed by more investigations of fungal diversity on Fagaceae in the future.

## Supplementary Material

XML Treatment for Phaeotubakia

XML Treatment for Phaeotubakia lithocarpicola

## References

[B1] Braun U Bien S Hantsch L Heuchert B (2014) *Tubakiachinensis* sp. nov. and a key to the species of the genus *Tubakia*. Schlechtendalia (Halle) 28: 23–28. 10.25673/90134

[B2] Braun U Nakashima C Crous PW Groenewald JZ Moreno-Rico O Rooney-Latham S Blomquist CL Haas J Marmolejo J (2018) Phylogeny and taxonomy of the genus *Tubakia* s. lat. Fungal Systematics and Evolution 1(1): 41–99. 10.3114/fuse.2018.01.04 32490362 PMC7259437

[B3] Carbone I Kohn LM (1999) A method for designing primer sets for speciation studies in filamentous ascomycetes. Mycologia 3(3): 553–556. 10.1080/00275514.1999.12061051

[B4] Doyle JJ Doyle JL (1990) Isolation of plant DNA from fresh tissue. Focus (San Francisco, Calif.) 12: 13–15.

[B5] Fan XL Bezerra JDP Tian CM Crous PW (2018) Families and genera of diaporthalean fungi associated with canker and dieback of tree hosts. Persoonia 40(1): 119–134. 10.3767/persoonia.2018.40.05 30504998 PMC6146645

[B6] Glass NL Donaldson GC (1995) Development of primer sets designed for use with the PCR to amplify conserved genes from filamentous ascomycetes. Applied and Environmental Microbiology 61(4): 1323–1330. 10.1128/aem.61.4.1323-1330.1995 7747954 PMC167388

[B7] Harrington TC McNew DL (2018) A re-evaluation of *Tubakia*, including three new species on *Quercus* and six new combinations. Antonie van Leeuwenhoek 111(7): 1003–1022. 10.1007/s10482-017-1001-9 29256000

[B8] Harrington TC McNew D Yun HY (2012) Bur oak blight, a new disease on *Quercusmacrocarpa* caused by *Tubakiaiowensis* sp. nov. Mycologia 104(1): 79–92. 10.3852/11-112 21937728

[B9] Jiang N Li J Piao CG Guo MW Tian CM (2018) Identification and characterization of chestnut branch-inhabiting melanocratic fungi in China. Mycosphere : Journal of Fungal Biology 9(6): 1268–1289. 10.5943/mycosphere/9/6/14

[B10] Jiang N Fan X Tian C Crous PW (2020) Reevaluating Cryphonectriaceae and allied families in Diaporthales. Mycologia 112(2): 267–292. 10.1080/00275514.2019.1698925 32091968

[B11] Jiang N Voglmayr H Piao CG Li Y (2021a) Two new species of *Diaporthe* (Diaporthaceae, Diaporthales) associated with tree cankers in the Netherlands. MycoKeys 85: 31–56. 10.3897/mycokeys.85.73107 34934385 PMC8648711

[B12] Jiang N Yang Q Fan XL Tian CM (2021b) *Micromelanconiskaihuiae* gen. et sp. nov., a new diaporthalean fungus from Chinese chestnut branches in southern China. MycoKeys 79: 1–16. 10.3897/mycokeys.79.65221 33958949 PMC8065008

[B13] Katoh K Rozewicki J Yamada KD (2019) MAFFT online service: Multiple sequence alignment, interactive sequence choice and visualization. Briefings in Bioinformatics 20(4): 1160–1166. 10.1093/bib/bbx108 28968734 PMC6781576

[B14] Liu SB Zhang ZX Liu RY Mu TC Zhang XG Li Z Xia JW (2022) Morphological and molecular identification of *Ellipsoidisporodochium* gen. nov. (Tubakiaceae, Diaporthales) in Hainan Province, China. Phytotaxa 552(4): 259–266. 10.11646/phytotaxa.552.4.3

[B15] Miller MA Pfeiffer W Schwartz T (2010) Creating the CIPRES Science Gateway for Inference of Large Phylogenetic Trees. Institute of Electrical and Electronics Engineers: New Orleans, LA, USA. 10.1109/GCE.2010.5676129

[B16] Morales-Rodríguez C Bastianelli G Aleandri M Doğmuş-Lehtijärvi HT Oskay F Vannini A (2021) Revealing novel interactions between oak and *Tubakia* species: Evidence of the efficacy of the sentinel arboreta strategy. Biological Invasions 23(12): 3749–3765. 10.1007/s10530-021-02614-4

[B17] Ronquist F Huelsenbeck JP (2003) MrBayes 3: Bayesian phylogenetic inference under mixed models. Bioinformatics (Oxford, England) 19(12): 1572–1574. 10.1093/bioinformatics/btg180 12912839

[B18] Rossman AY Farr DF Castlebury LA (2007) A review of the phylogeny and biology of the Diaporthales. Mycoscience 48(3): 135–144. 10.1007/S10267-007-0347-7

[B19] Senanayake IC Crous PW Groenewald JZ Maharachchikumbura SSN Jeewon R Phillips AJL Bhat DJ Perera RH Li QR Li WJ Tangthirasunun N Norphanphoun C Karunarathna SC Camporesi E Manawasighe IS Al-Sadi AM Hyde KD (2017) Families of Diaporthales based on morphological and phylogenetic evidence. Studies in Mycology 86(1): 217–296. 10.1016/j.simyco.2017.07.003 28947840 PMC5603113

[B20] Senanayake IC Jeewon R Chomnunti P Wanasinghe DN Norphanphoun C Karunarathna A Pem D Perera RH Camporesi E McKenzie EHC Hyde KD Karunarathna SC (2018) Taxonomic circumscription of Diaporthales based on multigene phylogeny and morphology. Fungal Diversity 93(1): 241–443. 10.1007/s13225-018-0410-z

[B21] Stamatakis A (2014) RAxML version 8: A tool for phylogenetic analysis and post-analysis of large phylogenies. Bioinformatics (Oxford, England) 30(9): 1312–1313. 10.1093/bioinformatics/btu033 24451623 PMC3998144

[B22] Swoffords DL 2003. PAUP*: Phylogenetic analysis using parsimony (* and other methods). Version 4.0b10. Sunderland, England.

[B23] Udayanga D Miriyagalla SD Manamgoda DS Lewers KS Gardiennet A Castlebury LA (2021) Molecular reassessment of diaporthalean fungi associated with strawberry, including the leaf blight fungus, *Paraphomopsisobscurans* gen. et comb. nov. (Melanconiellaceae). IMA Fungus 12(1): 1–21. 10.1186/s43008-021-00069-9 34158123 PMC8218473

[B24] Vilgalys R Hester M (1990) Rapid genetic identification and mapping of enzymatically amplified ribosomal DNA from several *Cryptococcus* species. Journal of Bacteriology 172(8): 4238–4246. 10.1128/jb.172.8.4238-4246.1990 2376561 PMC213247

[B25] Voglmayr H Rossman AY Castlebury LA Jaklitsch WM (2012) Multigene phylogeny and taxonomy of the genus *Melanconiella* (Diaporthales). Fungal Diversity 57(1): 1–44. 10.1007/s13225-012-0175-8

[B26] Voglmayr H Castlebury LA Jaklitsch WM (2017) *Juglanconis* gen. nov. on *Juglandaceae*, and the new family Juglanconidaceae (Diaporthales). Persoonia 38(1): 136–155. 10.3767/003158517X694768 29151630 PMC5645181

[B27] White TJ Bruns T Lee S Taylor J (1990) Amplification and direct sequencing of fungal ribosomal RNA genes for phylogenetics. PCR Protocols: A Guide to Methods and Applications 18: 315–322. 10.1016/B978-0-12-372180-8.50042-1

[B28] Zhang ZX Mu TC Liu SB Liu RY Zhang XG Xia JW (2021) Morphological and phylogenetic analyses reveal a new genus and two new species of Tubakiaceae from China. MycoKeys 84: 185–201. 10.3897/mycokeys.84.73940 34853547 PMC8629905

[B29] Zhu YQ Jiang N Dou ZP Xue H Piao CG Li Y (2022) Additions to the knowledge of *Tubakia* (Tubakiaceae, Diaporthales) in China. Journal of Fungi (Basel, Switzerland) 8(11): 1143. 10.3390/jof8111143 36354910 PMC9694254

